# Temporal trends and recent correlates in sedentary behaviours in Chinese children

**DOI:** 10.1186/1479-5868-8-93

**Published:** 2011-08-26

**Authors:** Zhaohui Cui, Louise L Hardy, Michael J Dibley, Adrian Bauman

**Affiliations:** 1 Sydney School of Public Health, Sydney Medical School, University of Sydney, Australia

**Keywords:** sedentary, screen, trends, children, adolescents, correlates, China

## Abstract

**Background:**

Sedentary behaviours (television, video and computer) are related to health outcomes independent of physical activity. Few studies have examined trends and correlates of sedentary behaviours among youth in developing nations. The current study is to examine temporal trends in sedentary behaviours and recent correlates of screen use in Chinese children during a period of economic transition.

**Methods:**

Secondary analysis of China Health and Nutrition Surveys. Cross-sectional data on sedentary behaviours including screen use among children aged 6-18 years from four surveys in 1997 (n = 2,469), 2000 (n = 1,838), 2004 (n = 1,382) and 2006 (n = 1,128). Temporal trends in screen use by socio-demographic characteristics were examined. The correlates of spending more than 2 hours per day on screen time in the most recent survey data (2006, n = 986) were analysed using survey logistic regression analysis.

**Results:**

Daily screen time significantly increased in each subgroup by age, sex and urban/rural residence, with the largest increase for urban boys aged 13-18 years from 0.5 hours to 1.7 hours, and for rural boys aged 6-12 years from 0.7 hours to 1.7 hours (p < 0.0001). Daily time in both homework and extracurricular cultural activity increased significantly from 2000 to 2004 but was stable from 2004 to 2006. Boys (OR: 1.41, 95%CI: 1.09 -1.82), having a TV in the bedroom (OR: 1.86, 95%CI: 1.15 - 3.01), having access to internet at home (OR: 1.93, 95%CI: 1.12 - 3.31) or at internet cafés (OR: 2.01, 95%CI: 1.21 - 3.34), or often watching TV with parents (OR: 2.27, 95%CI: 1.37 - 3.74) were all associated with being more likely to be high screen users (≥ 2 hours/day). While children aged 13-18 years (OR: 0.67, 95%CI: 0.46-0.97) were less likely to be high screen users. Children whose parents often have rules on their TV viewing (OR: 0.64, 95%CI: 0.37 - 1.10) were slightly but not significantly less likely to be high screen users.

**Conclusion:**

This study confirms sedentary behaviour has increased over the last decade in Chinese children. Efforts to ensure Chinese youth meet screen time guidelines include limiting access to screen technologies and encouraging parents to monitor their own screen time and to set limits on their child's screen time.

## Background

Concerns have been raised that sedentary behaviours among children and adolescents may displace the time available for participation in physical activity, resulting in overall lower energy expenditure [1] and that sedentary behaviours are related to chronic health outcomes independent of physical activity [2-4]. Evidence suggests that sedentary behaviours adopted during childhood track into adult life [5]. A better understanding of temporal changes and the correlates of sedentary behaviours among young people has important public health implications in reducing sedentary behaviours before they become lifestyle norms.

The most common sedentary activity among young people is screen time (i.e., watching television (TV), DVDs, videos, and computer use) [6,7]. Because of its popularity screen time has often been used as a proxy measure of sedentary behaviour among youth however this assumption has recently been challenged [8], and there is a need to measure a range of other, non screen related sedentary behaviours to truly understand sedentariness. To date, few studies reported other sedentary behaviours such as homework [6,7,9] and extracurricular cultural activity including reading, writing and drawing [7,9].

Much of the evidence on sedentary behaviours, including screen time, among young people comes from cross-sectional surveys undertaken in developed countries [7,10-13]. Conversely, little is known about sedentary behaviours among youth in developing nations, such as China, where ownership of technologies associated with sedentariness, such as televisions and computers, have dramatically increased in parallel with rapid socioeconomic growth over the last decade [14]. Further, the unique Chinese culture that has high expectations on children's academic performance may influence Chinese children's sedentary behaviours differently from those behaviours of children in developed countries.

For contemporary generations of Chinese children growing up in the fastest developing nation globally there is a greater risk of exposure to more sedentary activities. For this reason ascertaining the prevalence, correlates and temporal changes in sedentary behaviours, including screen time, have become a research priority. The lack of epidemiological information on sedentary behaviours from developing nations is a significant research gap. Information from developed nations is not necessarily applicable to countries under-going social and economic transition so there is a need to examine local data to inform appropriate interventions to change modifiable behaviours, such as sedentary activities. It is important to ascertain what the correlates of screen time are among Chinese children and adolescents and whether these differ from the correlates reported among Western youth. The purpose of this study was to examine the temporal trends and correlates of sedentary behaviours among youth using data from the four most recent China Health and Nutrition Surveys.

## Methods

### Study design

The China Health and Nutrition Surveys (CHNS) are an ongoing series of cross-sectional household surveys (with a nested longitudinal cohort) conducted since 1989 in eight provinces in China with an additional province included in surveys conducted since 2000 surveys [15]. The CHNS is not nationally representative; however the provinces selected provide significant variability in geography, economic development, and health indicators, so that they may be considered to be generally representative of China. Details of the survey design have been published elsewhere [15]. Briefly, four counties within each province (1 low-, 2 middle- and 1 high-income, based on per capita income reported by the National Bureau of Statistics) were randomly selected using a weighted sampling scheme. In addition, the provincial capital city along with a lower-income city was selected. Then the township capital and three villages within the counties and urban and suburban neighbourhoods within the cities were randomly selected. Finally, twenty households were randomly selected within each neighbourhood and all individuals within a household were interviewed by trained investigators.

The survey protocols, instruments, and the process for obtaining informed consent for this study were approved by institutional review committees of the University of North Carolina at Chapel Hill and the National Institute for Nutrition and Food Safety, Chinese Center for Disease Control and Prevention.

### Participants

Participants aged 6-18 years in the eight provinces that consecutively participated in all four surveys in 1997, 2000, 2004 and 2006 were included in the analysis. Older children were asked survey questions directly. Parents were present for interviews with children younger than 10 years and often assisted the child with answering, or answered the questions directly as a proxy for the child.

### Measurements

Demographic information included the child's date of birth, sex, and family annual income. Per capita family annual income in Chinese currency (RMB) was obtained by dividing family annual income by household size. The per capita family annual income in each survey was inflated to values in 2009 by adjusting for consumer price index and then categorized into tertiles as high, medium and low income by urban and rural areas. Other information collected included the child's self-perceived weight status, whether there was a TV in the child's bedroom, internet access, family's rules on child's TV viewing, and parent-child TV co-viewing.

### Sedentary time

Information on children's sedentary time was collected from children aged 6-18 years for the first time in 1997. In 1997, the questionnaire asked the child to report the usual time spent watching TV/video tapes. Since 2000, time spent doing homework, and extracurricular cultural activity including reading, writing and drawing was added to the CHNS. Since 2004, new questions asked about time spent watching VCDs and DVDs, playing video games and using computer as the private ownership of these technologies increased during this period [14]. For the analysis of correlates of screen time, screen time was categorized into two groups (less than 2 hour per day and 2 hours per day or more) based on the international screen time recommendations [16,17].

### Statistical analysis

Participants are divided into two age groups (i.e. 6-12 years and 13-18 years) because they generally move into middle schools after 12 years old. The median time spent on different sedentary activities per day in each survey was calculated by age group, sex, urban or rural residence. In each survey since 2000, the participants consist of two parts: newly recruited and those have been measured in previous surveys (i.e. nested cohort). There was no difference in the median times of sedentary activities across survey years between the newly-recruited and the nested cohort participants. Thus, median time and its trends from all participants were reported in this paper. The Kruskal-Wallis one-way analysis of variance was used to examine the difference in sedentary time between surveys in each subgroup.

In the analysis of correlates of screen time, survey logistic regression analysis was applied to account for intraclass correlation incurred by stratified multi-stage cluster random sampling, with "province" as strata variable and "county" as primary sampling unit. Stepwise elimination process was applied for model choice. Sociodemographic variables including age, sex, urban or rural residence and per capita family annual income were retained in the model. All the analyses were conducted using SAS (Version 9; SAS Institute, Cary, NC, USA).

## Results

Table 1 summarises the characteristics of the participants in 1997, 2000, 2004 and 2006. In the four surveys, there were 2,469, 1,838, 1,382 and 1,128 children, respectively. The mean ages across surveys ranged from 11.7 years to 12.0 years (p < 0.01). There was no significant difference in the proportion of urban or rural residence. The per capita family annual income increased from 4,389 RMB to 8,453 RMB in urban areas (p < 0.01) and from 3,178 RMB to 5,187 RMB in rural areas (p < 0.0001) between 1997 and 2006. The prevalence of spending more than 2 hours per day on screen time increased from approximately 10% in 1997 to approximately 40% in 2006 in both urban and rural areas (p < 0.0001).

**Table 1:** Characteristics of the study population from 1997 to 2006: China Health and Nutrition Surveys

	Survey year			
	
	1997	2000	2004	2006
*n*	2469	1838	1382	1128
Age (yrs, mean ± SD)**	11.7 ± 3.1	12.0 ± 2.9	12.0 ± 3.2	11.7 ± 3.2
Male (%)	52.5	54.0	52.6	53.1
Urban (%)	29.6	29.4	29.4	28.9
*Per capita family annual income (RMB^§^, mean ± SD)^†^*				
Urban**	4389 ± 3090	5806 ± 4957	6980 ± 6368	8453 ± 8570
Rural***	3178 ± 2605	3728 ± 3042	4799 ± 4781	5187 ± 7820
*Prevalence of screen time ≥ 2 hrs/d (%, Urban)*				
Boys aged 6-12 yrs*	13.3	16.4	31.7	36.7
Boys aged 13-18 yrs***	11.2	11.1	42.9	44.3
Girls aged 6-12 yrs***	13.5	11.3	33.7	27.2
Girls aged 13-18 yrs***	9.7	8.5	25.3	34.7
*Prevalence of screen time ≥ 2 hrs/d (%, Rural)*				
Boys aged 6-12 yrs***	9.4	11.5	30.3	46.7
Boys aged 13-18 yrs***	5.7	7.6	22.9	32.5
Girls aged 6-12 yrs***	10.2	11.6	28.4	42.3
Girls aged 13-18 yrs***	3.0	8.3	16.7	24.6

*P < 0.01. **P < 0.001. ***P < 0.0001.

^§ ^RMB denotes Chinese currency.

^†^The per capita family annual income in each survey year was inflated to values in 2009 by adjusting for consumer price index.

As shown in Table 2, median screen time increased significantly in each subgroup by age, sex and region of residence from 1997 to 2006. The most significant increase in daily screen time in urban areas was among boys aged 13-18 years from 0.5 hour in 1997 and 2000, to 1.4 hours in 2004 and to 1.7 hours in 2006 (p < 0.0001), and in rural areas for boys aged 6-12 years daily screen time increased from 0.7 hour in 1997 and 2000, to 1.3 hours in 2004 and to 1.7 hours in 2006 (p < 0.0001).

**Table 2:** Median daily hours for screen time, home work and extracurricular reading, writing and drawing among Chinese youth by sex and age from 1997 to 2006^§^

	Urban				Rural			
		
	1997	2000	2004	2006	1997	2000	2004	2006
*Screen time (hrs/d)*^†^								
Boys aged 6-12 yrs***	0.9	0.6	1.3	1.3	0.7	0.7	1.3	1.7
Boys aged 13-18 yrs***	0.5	0.5	1.4	1.7	0.4	0.4	1.0	1.3
Girls aged 6-12 yrs***	0.9	0.6	1.3	1.2	0.7	0.7	1.3	1.6
Girls aged 13-18 yrs***	0.5	0.5	1.2	1.3	0.3	0.4	1.0	1.1
*Homework (hrs/d)*								
Boys aged 6-12 yrs	-	0.6**	1.0	0.9	-	0.6***	1.0	1.0
Boys aged 13-18 yrs***	-	0.7	1.5	1.7	-	0.8	1.0	1.2
Girls aged 6-12 yrs***	-	0.7	1.2	1.2	-	0.6	1.0	1.0
Girls aged 13-18 yrs***	-	0.9	1.6	1.9	-	0.9	1.5	1.3
*Extracurricular reading, writing and drawing*						-		
Boys aged 6-12 yrs	-	0.1***	0.4	0.5	-	0.1**	0.2	0.3
Boys aged 13-18 yrs***	-	0.1	0.5	0.4	-	0.1	0.4	0.4
Girls aged 6-12 yrs	-	0.3*	0.5	0.5	-	0.1	0.0	0.3
Girls aged 13-18 yrs***	-	0.2	0.5	0.5	-	0.1	0.6	0.5

^§ ^The NPAR1WAY Procedure was used to examine the difference between survey years.

^† ^Screen time denotes time on watching TV, videotapes in 1997 and 2000, and on TV, videotapes, VCDs, DVDs, video games, computer usage in 2004 and 2006.

*P < 0.01. **P < 0.001. ***P < 0.0001.

Table 2 also shows a significant increase in time engaged in homework and extracurricular cultural activity time from 2000 to 2006. Although significant increases were found in each subgroup by age, sex and urban or rural residence in time on homework between 2000 and 2004, the time spent on homework was stable between 2004 and 2006 in most subgroups with the exception of an overall increase among participants aged 13-18 years and urban girls aged 13-18 years. A similar universal increasing trend was found in extracurricular cultural activity between 2000 and 2004, but it was stable in all subgroups between 2004 and 2006.

Table 3 presents the correlates of spending more than 2 hours per day in screen time from the analysis in the most recent CHNS (i.e., 2006). Children aged 13-18 years were 33% less likely (OR: 0.67, 95%CI: 0.46-0.97) to spend more than 2 hours per day on screen behaviours compared with those aged 6-12 years. Boys were 1.41 times (OR: 1.41, 95%CI: 1.09 -1.82) more likely to exceed the 2 hour per day screen time limit compared with girls. Children with a TV in their bedrooms were 86% more likely (OR: 1.86, 95%CI: 1.15 - 3.01) to spend more than 2 hours per day on screen time than those without a TV in their bedroom. Similarly, children with access to internet at home or at internet cafés were twice as likely to exceed the 2 hour per day screen time limit as those without access to internet. Compared with those children who seldom watched TV with their parents, children who often watched TV with their parents were 2.27 times (OR: 2.27, 95%CI: 1.37 - 3.74) more likely to spend more than 2 hours per day on screen. Children whose parents often set rules about their TV viewing were 36% less likely (OR: 0.64, 95%CI: 0.37 - 1.10) to spend more than 2 hours per day on screen time compared with those whose parents seldom had rules about their TV viewing although it was not statistically significant (*P *= 0.075).

**Table 3:** Correlates of spending more than 2 hours/day of screen time outside school in Chinese children in 2006 (n = 986)^§^

	≥ 2 hrs/d (%)	Unadjusted OR	Adjusted OR
*Age*			
6-12 yrs	42.9	1.0	1.0
13-18 yrs	32.4	0.64 (0.45-0.91)	0.67(0.46-0.97)
*Sex*			
Girls	34.3	1.0	1.0
Boys	43.9	1.49 (1.17-1.91)	1.41(1.09-1.82)
*Region*			
Rural	41.9	1.0	
Urban	33.2	0.69 (0.39-1.23)	0.62(0.36-1.09)
*Per capita family income*			
Low	40.7	1.0	1.0
Medium	41.0	1.01 (0.67-1.54)	1.02(0.68-1.53)
High	36.5	0.84 (0.55-1.27)	0.84(0.56-1.26)
*Self-perceived weight status*			
Healthy weight	39.0	1.0	
Underweight	44.7	1.27(0.77-2.08)	
Overweight/obesity	33.8	0.80 (0.45-1.41)	
*TV in child's bedroom*			
No	37.6	1.0	1.0
Yes	51.7	1.77 (1.09-2.87)	1.86(1.15-3.01)
*Internet access in internet cafés*			
No	37.8	1.0	1.0
Yes	48.3	1.54 (0.91-2.60)	2.01(1.21-3.34)
*Internet access at home*			
No	38.9	1.0	1.0
Yes	44.9	1.28 (0.77-2.14)	1.93(1.12-3.31)
*Internet access at relative/friend's home*			
No	39.4	1.0	
Yes	40.0	1.03 (0.60-1.75)	
*Watching TV with parent(s)*			
Seldom	31.4	1.0	1.0
Sometimes	36.9	1.28 (0.88-1.87)	1.26(0.86-1.86)
Often	51.3	2.30 (1.44-3.69)	2.27(1.37-3.74)
*Family's rules about TV viewing*			
Seldom	41.4	1.0	1.0
Sometimes	41.3	0.99 (0.66-1.49)	1.02(0.69-1.50)
Often	30.4	0.62 (0.37-1.04)	0.64(0.37-1.10)
*Family ask you engage in physical activity*			
Don't care	39.6	1.0	
More physical activity	38.3	0.95 (0.66-1.36)	
Less physical activity	46.0	1.30 (0.65-2.60)	

^§ ^Stepwise backward elimination SURVEYLOGISTIC regression was used to model the correlates initially from a full model with all variables in the left column in the Table.

## Discussion

The findings from this study showed significant increases in screen time, homework and extracurricular cultural activity across the survey period for all ages among boys and girls. Further, there was strong evidence to show that the prevalence of Chinese youth who exceed the recommended screen time guideline increased significantly over the last decade. Using the most recent survey data (i.e., 2006) the study also found that the correlates of spending more than 2 hours per day on screen time were similar to those reported among Western youth. The odds of high screen time among Chinese youth were strongly associated with younger aged boys, participants with a TV in the bedroom [18-21], having access to internet at internet cafés or at home, co-viewing TV with their parents [22,23] and having no parental rules about their TV viewing [18,23-26]. Few studies have examined sedentary behaviour in Chinese youth and this study makes a unique contribution to our understanding of national trends and current correlates of sedentary behaviours and screen time among Chinese children and adolescents.

Only a few studies have assessed temporal trends in screen time [5,27,28] among children in developed nations, none from developing countries, and no studies have examined temporal trends of non screen time sedentary activities such as homework and other extracurricular cultural activities. The present analysis found a significant increase in both screen time and the proportion of young Chinese spending 2 or more hours per day on screen time over the last decade. The trends in our analysis are consistent with previous studies from developed countries [5,27] but reveal more rapid increases than previously reported. Our findings coincide with the economic transitioning which is occurring in China. During this period in China there was a rapid increase in the family income, in the ownership of televisions, video players and computers [14], and in the prevalence of child obesity [29]. Two studies which have examined sedentary trends among youth, using screen time as a proxy measure, reported decreasing or stable trends in TV time, probably because TV time was substituted by other multiple new entertainment options [28,30].

Studies in Chinese adults suggested that the decline in physical activity were associated with urbanization [31,32]. Increases in urbanization in many developing nations, including China, have associated with a rapid transition to a more sedentary lifestyle through the acquisition of new technologies [32-34]. In countries which are undergoing rapid social transition, interventions to reduce sedentary time and to promote physical activity in different settings (school, home, transport, neighbourhood recreation facilities etc.) should be implemented to counter the negative health impact of urbanization [31,35].

Studies among Western youth in the US [36], UK [7], France [27], Canada [9] and Australia [6,13] conducted between 1999 and 2006 have reported that approximately two-thirds of youth exceed the screen time guidelines and that the average screen time among Western youth was approximately 2.5 hours per day. In Mexico City, youth watched TV for 2.4 hours per day and videos for 0.9 hours per day in 1997, respectively, with a 40% of youth exceeding the screen time guidelines [37]. Another developing country from South America, Brazil [38], reported a 2.7 hours per day of screen time among their youth in 2005-2006. These findings are in contrast to the current study findings and other findings from Asian based studies which indicate screen time is lower among youth in a range of Asian countries compared with Western and South American youth. However screen use in Asia was related to the economic prosperity of the country. For example the prevalence in this study of exceeding the screen time guideline in 2006 was between 25% - 45% with a median screen time of 85 minutes per day, consistent with a previous study in China [39], while in Vietnam the prevalence was 19.1% [40]. In developed Asian nations such as Japan [41], the prevalence of exceeding the screen time guideline was 47.1% and in Korea [42], the average youth watches television for 2.6 hours per day and an additional 2.3 hours per day on a computer. Early comparisons indicated that Filipino youth watched substantially more hours of television than did Chinese youth in 1997 (1.9 ± 1.2 vs. 0.5 ± 0.6 hour/day) [43].

Of the few studies [7,9] which have examined other sedentary behaviours, such as homework, this study found that the time Chinese children reported spending on homework was much higher than the time reported by children in developed countries. In the current study, two thirds of Chinese children (64.5%) in 2006 spent one or more hours per day on homework which was three times higher than youth from Canada (22.9% in 2005-2006) [9], the UK (around 30% in 2002) [7] and Australia [6]. This difference in time on homework may due to a more academic-oriented culture in China than in Western countries, which is supported by literature report that Asian Australian children spent more time on homework than Caucasian children [9].

The decrease in the prevalence of spending more than 2 hours per day on screen time with age among rural youth in this study was inconsistent with previous cross-sectional [13,25,26,44] and longitudinal [5,45] studies that indicated screen time increases with age. This decline with age in screen time may be explained by the unique Chinese setting in which most rural high schools are boarding schools where adolescents have limited access to screen facilities. Also, it may be because adolescents attending high schools have more extracurricular academic activities like homework and night classes, as our analysis showed that older children spent more time on homework. Also, adolescents may shift screen time to non-screen sedentary behaviours, like talking with friends and listening to music [12]. In urban areas, the proportion of children exceeding screen time guidelines shifted from younger children to older children. This may be because urban adolescents have had progressively greater accessibility to screen-based devices [25]. Consistent with previous studies [12,13,25,26,45], our study found that boys reported more hours of sedentary activity than girls [28,45]. This may be partly explained by girls taking part in more varied activities other than screen behaviours, such as homework and extracurricular cultural activities as found in our study and in previous studies [6,9].

Consistent with previous reports [20,21,46,47], this study found that the presence of a TV in the child's bedroom had a strong positive association with high screen use among children and adolescents. Access to internet at home or at internet cafés also showed a positive association with high screen use. A further analysis found that only a small proportion (4.4%) of rural children had internet access at home, indicating that the internet time of rural children may be mainly attributed to internet café usage. The strong association between these environmental factors with screen time suggests that child's screen time could be reduced by relatively simple changes such as removing the TV from the child's bedroom or limiting their access to the internet.

Although it might be unreasonable to create an electronic media-free home environment, parental rules on children's use of screen technologies may restrict the screen time of children without loss of the benefits of access to these media. Our results demonstrated that parental rules on youths' TV viewing were associated with higher screen time, although it was not statistically significant, which is consistent with previous study findings [23-26,46]. Interestingly, this association was stronger when rules were 'often' present compared with 'sometimes' present, which suggests that a threshold may exist for the intensity of rules.

Consistent with previous studies [22,26], our study found a strong positive association between parent-child co-viewing TV and the child's screen time. These findings indicate that parents may influence their child's screen behaviours through role modelling and therefore interventions that target both child and parental TV viewing may be more effective in reducing children's screen use [26].

### Limitations and strengths

One of the strengths of this study is the large population, selected to represent Chinese youth in rural and urban China. In addition, the sampling and data collection methods were identical in each survey. Also, short intervals between surveys allowed us to carefully examine the trends in sedentary behaviours in China. One of the limitations of the study is that the cross-sectional data limits inference of causality in the analysis of correlates of high screen use. Additionally, over time the questions on the CHNS changed to reflect the emerging screen technologies in China. For example, questions on computer time were added in 2004 as household ownership of computers changed. In China there were 9.7 computers per 100 urban households in 2000, but this had increased to 33.1 computers per 100 urban households by 2004 [14]. The addition of VCD and DVD use to the questionnaire slightly lagged behind the increase in ownership of these devices in China, which moved from 7.9 in 1997 to 37.5 in 2000 per 100 urban households [14]. The overall trends in screen time between 2000 and 2004 were not influenced by this lag in introducing questions about use of VCD and DVD because of the very low proportion (14.2%) and average time of usage (0.1 hour/day) in the study population in 2000.

Another limitation is that the CHNS data is not weighted because the sampling frame was not available in 1989 when the sites were selected, thus the findings may not be generalized to the population from which the sample was drawn. However, the unweighted analysis may not change the temporal trends in the sedentary behaviours in this population considering the comparable sample characteristics of sex and region of residence between surveys and the stratified analysis by age, sex and region of residence. Finally, although non-independent samples across survey years may be not ideal for the examination of temporal trends, our analysis found similar median times and temporal trends in sedentary activities with those obtained from the separate analysis using only newly recruited participants.

## Conclusion

This study shows that sedentary behaviour and screen time increased among a large population sample of Chinese children aged 6-18 years between 1997 and 2006. This increase occurred during a time of economic and social transition, where access to screen technologies increased. It would appear that as China continues to prosper many Chinese children are potentially at risk of adopting sedentary lifestyles typically associated with Western youth. Efforts should aim to ensure Chinese youth meet screen time guidelines, including limiting access to screen technologies and encouraging parents to monitor their own screen time and to set limits on their child's screen time.

## List of abbreviation

CHNS: China Health and Nutrition Survey; TV: Television

## Competing interests

The authors declare that they have no competing interests.

## Authors' contributions

ZC performed the statistical analysis and drafted the manuscript. LH guided the statistical analysis and critically revised the manuscript. MD provided suggestions in the statistical analysis and approved the final manuscript. AB supervised the analysis and revised the manuscript. All authors read and approved the final manuscript.

## References

[B1] British Heart Foundation Couch kids: The continuing epidemic 2004 London: British Heart Foundation

[B2] Ekelund U Brage S Froberg K Harro M Anderssen SA Sardinha LB Riddoch C Andersen LB TV viewing and physical activity are independently associated with metabolic risk in children: the European Youth Heart Study PLoS medicine 2006 3 e488 10.1371/journal.pmed.0030488 17194189 PMC1705825

[B3] Hardy LL Dobbins TA Denney-Wilson EA Okely AD Booth ML Sedentariness, small-screen recreation, and fitness in youth Am J Prev Med 2009 36 120 125 10.1016/j.amepre.2008.09.034 19135904

[B4] Hardy LL Denney-Wilson E Thrift AP Okely AD Baur LA Screen time and metabolic risk factors among adolescents Arch Pediatr Adolesc Med 2010 164 643 649 10.1001/archpediatrics.2010.88 20603465

[B5] Nelson MC Neumark-Stzainer D Hannan PJ Sirard JR Story M Longitudinal and secular trends in physical activity and sedentary behavior during adolescence Pediatrics 2006 118 e1627 1634 10.1542/peds.2006-0926 17142492

[B6] Hardy LL Dobbins T Booth ML Denney-Wilson E Okely AD Sedentary behaviours among Australian adolescents Aust N Z J Public Health 2006 30 534 540 10.1111/j.1467-842X.2006.tb00782.x 17209269

[B7] Gorely T Biddle SJ Marshall SJ Cameron N The prevalence of leisure time sedentary behaviour and physical activity in adolescent boys: An ecological momentary assessment approach Int J Pediatr Obes 2009 4 289 298 10.3109/17477160902811181 19922044

[B8] Biddle SJ Gorely T Marshall SJ Is television viewing a suitable marker of sedentary behavior in young people? Ann Behav Med 2009 38 147 153 10.1007/s12160-009-9136-1 19809858

[B9] Leatherdale ST Wong SL Modifiable characteristics associated with sedentary behaviours among youth Int J Pediatr Obes 2008 3 93 101 18465435 10.1080/17477160701830879

[B10] Guthold R Cowan MJ Autenrieth CS Kann L Riley LM Physical activity and sedentary behavior among schoolchildren: a 34-country comparison J Pediatr 2010 157 43 49 10.1016/j.jpeds.2010.01.019 20304415

[B11] Sisson SB Church TS Martin CK Tudor-Locke C Smith SR Bouchard C Earnest CP Rankinen T Newton RL Katzmarzyk PT Profiles of sedentary behavior in children and adolescents: the US National Health and Nutrition Examination Survey, 2001-2006 Int J Pediatr Obes 2009 4 353 359 10.3109/17477160902934777 19922052 PMC2891818

[B12] Olds T Wake M Patton G Ridley K Waters E Williams J Hesketh K How do school-day activity patterns differ with age and gender across adolescence? J Adolesc Health 2009 44 64 72 10.1016/j.jadohealth.2008.05.003 19101460

[B13] Hardy LL Dobbins TA Denney-Wilson EA Okely AD Booth ML Descriptive epidemiology of small screen recreation among Australian adolescents J Paediatr Child Health 2006 42 709 714 10.1111/j.1440-1754.2006.00956.x 17044899

[B14] National Bureau of Statistics of China China Statistics Yearbook 2007 2007 Beijing: China Statistics Press

[B15] Popkin BM Du S Zhai F Zhang B Cohort Profile: The China Health and Nutrition Survey--monitoring and understanding socio-economic and health change in China, 1989-2011 Int J Epidemiol 2010 39 1435 1440 10.1093/ije/dyp322 19887509 PMC2992625

[B16] The Australian College of Paediatrics: Policy statement. Children's television J Paediatr Child Health 1994 30 6 8 8148193

[B17] Strong WB Malina RM Blimkie CJ Daniels SR Dishman RK Gutin B Hergenroeder AC Must A Nixon PA Pivarnik JM Evidence based physical activity for school-age youth J Pediatr 2005 146 732 737 10.1016/j.jpeds.2005.01.055 15973308

[B18] Wiecha JL Sobol AM Peterson KE Gortmaker SL Household television access: associations with screen time, reading, and homework among youth Ambul Pediatr 2001 1 244 251 10.1367/1539-4409(2001)001<0244:HTAAWS>2.0.CO;2 11888409

[B19] Saelens BE Sallis JF Nader PR Broyles SL Berry CC Taras HL Home environmental influences on children's television watching from early to middle childhood J Dev Behav Pediatr 2002 23 127 132 10.1097/00004703-200206000-00001 12055494

[B20] Gorely T Marshall SJ Biddle SJ Couch kids: correlates of television viewing among youth Int J Behav Med 2004 11 152 163 10.1207/s15327558ijbm1103_4 15496343

[B21] van Zutphen M Bell AC Kremer PJ Swinburn BA Association between the family environment and television viewing in Australian children J Paediatr Child Health 2007 43 458 463 10.1111/j.1440-1754.2007.01111.x 17535176

[B22] Hardy LL Baur LA Garnett SP Crawford D Campbell KJ Shrewsbury VA Cowell CT Salmon J Family and home correlates of television viewing in 12-13 year old adolescents: the Nepean Study Int J Behav Nutr Phys Act 2006 3 24 10.1186/1479-5868-3-24 16961929 PMC1594572

[B23] Hoyos Cillero I Jago R Systematic review of correlates of screen-viewing among young children Prev Med 2010 51 3 10 10.1016/j.ypmed.2010.04.012 20417227

[B24] Krosnick JA Anand SN Hartl SP Psychosocial Predictors of Heavy Television Viewing Among Preadolescents and Adolescents Basic Appl Soc Psych 2003 25 87 110 10.1207/S15324834BASP2502_1

[B25] Hoyos Cillero I Jago R Sociodemographic and home environment predictors of screen viewing among Spanish school children J Public Health (Oxf) 2010 10.1093/pubmed/fdq087 PMC3307230 21047871

[B26] Hoyos Cillero I Jago R Sebire S Individual and social predictors of screen-viewing among Spanish school children Eur J Pediatr 2011 170 93 102 10.1007/s00431-010-1276-6 20814697

[B27] Lioret S Touvier M Dubuisson C Dufour A Calamassi-Tran G Lafay L Volatier JL Maire B Trends in child overweight rates and energy intake in France from 1999 to 2007: relationships with socioeconomic status Obesity 2009 17 1092 1100 10.1038/oby.2008.619 19148118

[B28] Samdal O Tynjala J Roberts C Sallis JF Villberg J Wold B Trends in vigorous physical activity and TV watching of adolescents from 1986 to 2002 in seven European Countries Eur J Public Health 2007 17 242 248 10.1093/eurpub/ckl245 17071949

[B29] Cui Z Huxley R Wu Y Dibley MJ Temporal trends in overweight and obesity of children and adolescents from nine Provinces in China from 1991-2006 Int J Pediatr Obes 2010 5 365 374 10.3109/17477166.2010.490262 20836722

[B30] Centers for Disease Control and Prevention Youth Risk Behavior Surveillance System 2011 http://www.cdc.gov/HealthyYouth/yrbs/index.htm Accessed March 23

[B31] Ng SW Norton EC Popkin BM Why have physical activity levels declined among Chinese adults? Findings from the 1991-2006 China Health and Nutrition Surveys Soc Sci Med 2009 68 1305 1314 10.1016/j.socscimed.2009.01.035 19232811 PMC2731106

[B32] Monda KL Gordon-Larsen P Stevens J Popkin BM China's transition: the effect of rapid urbanization on adult occupational physical activity Soc Sci Med 2007 64 858 870 10.1016/j.socscimed.2006.10.019 17125897 PMC2753984

[B33] Popkin BM Gordon-Larsen P The nutrition transition: worldwide obesity dynamics and their determinants Int J Obes 2004 28 Suppl 3 S2 9 10.1038/sj.ijo.0802804 15543214

[B34] Popkin BM The nutrition transition and obesity in the developing world J Nutr 2001 131 871S 873S 11238777 10.1093/jn/131.3.871S

[B35] Popkin BM The nutrition transition in the developing world Dev Policy Rev 2003 21 581 597 10.1111/j.1467-8659.2003.00225.x

[B36] Eisenmann JC Bartee RT Wang MQ Physical activity, TV viewing, and weight in U.S. youth: 1999 Youth Risk Behavior Survey Obes Res 2002 10 379 385 10.1038/oby.2002.52 12006637

[B37] Hernandez B Gortmaker SL Colditz GA Peterson KE Laird NM Parra-Cabrera S Association of obesity with physical activity, television programs and other forms of video viewing among children in Mexico city Int J Obes 1999 23 845 854 10.1038/sj.ijo.0800962 10490786

[B38] de Oliveira TC da Silva AA dos Santos Cde J Silva JS da Conceicao SI Physical activity and sedentary lifestyle among children from private and public schools in Northern Brazil Rev Saude Publica 2010 44 996 1004 10.1590/S0034-89102010000600003 21107499

[B39] Tudor-Locke C Ainsworth BE Adair LS Du S Popkin BM Physical activity and inactivity in Chinese school-aged youth: the China Health and Nutrition Survey Int J Obes Relat Metab Disord 2003 27 1093 1099 10.1038/sj.ijo.0802377 12917716

[B40] Tang KH Nguyen HH Dibley MJ Sibbritt DW Phan NT Tran TM Factors associated with adolescent overweight/obesity in Ho Chi Minh city Int J Pediatr Obes 2010 5 396 403 10.3109/17477160903540735 20233155

[B41] Sun Y Sekine M Kagamimori S Lifestyle and overweight among Japanese adolescents: the Toyama Birth Cohort Study J Epidemiol 2009 19 303 310 10.2188/jea.JE20080095 19776497 PMC3924099

[B42] Kang HT Lee HR Shim JY Shin YH Park BJ Lee YJ Association between screen time and metabolic syndrome in children and adolescents in Korea: the 2005 Korean National Health and Nutrition Examination Survey Diabetes Res Clin Pract 2010 89 72 78 10.1016/j.diabres.2010.02.016 20299120

[B43] Tudor-Locke C Ainsworth BE Adair LS Du S Lee N Popkin BM Cross-sectional comparison of physical activity and inactivity patterns in Chinese and Filipino youth Child Care Health Dev 2007 33 59 66 10.1111/j.1365-2214.2006.00612.x 17181754

[B44] Kristjansdottir G Vilhjalmsson R Sociodemographic differences in patterns of sedentary and physically active behavior in older children and adolescents Acta Paediatr 2001 90 429 435 11332936

[B45] Brodersen NH Steptoe A Boniface DR Wardle J Trends in physical activity and sedentary behaviour in adolescence: ethnic and socioeconomic differences Br J Sports Med 2007 41 140 144 10.1136/bjsm.2006.031138 17178773 PMC2465219

[B46] Wiecha JL Sobol AM Peterson KE Gortmaker SL Household television access: associations with screen time, reading, and homework among youth Ambul Pediatr 2001 1 244 251 10.1367/1539-4409(2001)001<0244:HTAAWS>2.0.CO;2 11888409

[B47] Saelens BE Sallis JF Nader PR Broyles SL Berry CC Taras HL Home environmental influences on children's television watching from early to middle childhood J Dev Behav Pediatr 2002 23 127 132 10.1097/00004703-200206000-00001 12055494

